# A Novel Method for the Discrimination of Semen Arecae and Its Processed Products by Using Computer Vision, Electronic Nose, and Electronic Tongue

**DOI:** 10.1155/2015/753942

**Published:** 2015-08-26

**Authors:** Min Xu, Shi-Long Yang, Wei Peng, Yu-Jie Liu, Da-Shuai Xie, Xin-Yi Li, Chun-Jie Wu

**Affiliations:** ^1^College of Pharmacy, Chengdu University of Traditional Chinese Medicine, No. 1166, Liutai Road, Wenjiang District, Chengdu 611137, China; ^2^The Key Laboratory of Technology of Chinese Medicine Processing, State Administration of Traditional Chinese Medicine, No. 1166, Liutai Road, Wenjiang District, Chengdu 611137, China

## Abstract

Areca nut, commonly known locally as Semen Arecae (SA) in China, has been used as an important Chinese herbal medicine for thousands of years. The raw SA (RAW) is commonly processed by stir-baking to yellow (SBY), stir-baking to dark brown (SBD), and stir-baking to carbon dark (SBC) for different clinical uses. In our present investigation, intelligent sensory technologies consisting of computer vision (CV), electronic nose (E-nose), and electronic tongue (E-tongue) were employed in order to develop a novel and accurate method for discrimination of SA and its processed products. Firstly, the color parameters and electronic sensory responses of E-nose and E-tongue of the samples were determined, respectively. Then, indicative components including 5-hydroxymethyl furfural (5-HMF) and arecoline (ARE) were determined by HPLC. Finally, principal component analysis (PCA) and discriminant factor analysis (DFA) were performed. The results demonstrated that these three instruments can effectively discriminate SA and its processed products. 5-HMF and ARE can reflect the stir-baking degree of SA. Interestingly, the two components showed close correlations to the color parameters and sensory responses of E-nose and E-tongue. In conclusion, this novel method based on CV, E-nose, and E-tongue can be successfully used to discriminate SA and its processed products.

## 1. Introduction

Areca nut, commonly known locally as Semen Arecae (SA) in China, is a product (dried seed preparation) from* Areca catechu* (Areca palm tree) that has been used as an important Chinese herbal medicine for thousands of years. The raw SA (RAW) is commonly processed by stir-baking to yellow (SBY), stir-baking to dark brown (SBD), and stir-baking to carbon dark (SBC) for different clinical uses. Plenty of pharmacological investigations have demonstrated that SA possessed a wide range of bioactivities including antiparasitic, anti-inflammatory, and analgesic effects and effects on digestive system [1].

Although the other three processed products of SA are stir-baked from RAW, the efficacies are very different. Thus, it is very crucial to discriminate them before clinical use. Currently, judgment of the degree of stir-baking of SA can be summarized as two steps. Firstly, it was evaluated according to the characters of color, odor, or taste by trained specialists, which is called human sensory analysis. Then, the indicative components were determined by HPLC. Although HPLC is effective to both qualitative and quantitative analysis, some disadvantages are still existing in HPLC analysis, including cumbersome operation, resource wasting, and reagent pollution. In addition, human sensory analysis is a simple, rapid, and nondestructive method, but it is highly dependent on subjective judgment and might be swayed by physical and environmental factors [2]. There is no doubt that the characters are closely related to the inherent quality of the sample [2–4]. Therefore, in order to overcome these weaknesses of the above-mentioned analysis methods, we proposed to use intelligent sensory technologies to discriminate SA and its processed products.

Intelligent sensory technologies that imitate the characteristics of human senses, such as the senses of sight, smell, and taste, consist of computer vision (CV), electronic nose (E-nose), and electronic tongue (E-tongue). CV is a novel technology for recognizing objects and extracting and analyzing quantitative information from digital images [5], and it is an imitation of human vision system, which is highly adaptable to different conditions. In addition, CV can present significant objective information about color and convert color to reproducible numerical values. A number of previous researches have reported that CV is an effective way for color measurement [4–8]. E-nose is a machine designed to detect complex odors based on an array of metal oxide sensors. The sensor array consists of broadly tuned (nonspecific) sensors, made of a variety of odor-sensitive biological or chemical materials [9]. Actually, representative odor fingerprints are obtained and employed to construct a database and train a pattern recognition system, such that later unknown odors can subsequently be classified and identified. Recently, E-nose has been employed in discrimination of traditional medicine, food and agroproducts, and so forth, such as musk [3],* Asteraceae *plants [10], coffee [11], fruit [12], and pork [13]. E-tongue can be considered as an analytical instrument that artificially reproduces the taste sensation [14]. This device typically includes an array of high stability, cross-sensitivity, and ion-selective sensors coupled to chemometric processing used to characterize complex liquid samples [15–17]. E-tongue is capable of analyzing and discriminating various products, such as wine [18], honey [19], tea [20],* Perilla frutescens *[21],* Fritillaria *[22], herbal extracts, and throat lozenges [23]. In addition, E-nose and E-tongue are often combined together for edible products and pharmaceutical uses [9, 24].

As mentioned above, CV, E-nose, and E-tongue technologies have been successfully applied in the discrimination of different products as effective methods. However, there is no report regarding the application of the aforementioned technologies for the discrimination of SA and its processed products. Therefore, this research sought to develop a rapid, objective, and accurate detection method to discriminate SA and its processed products using CV, E-nose, and E-tongue coupled with chemometrics. Furthermore, the components significantly related to the stir-baking processes were determined, and the correlations between components and electronic values of intelligent sensory technologies were investigated.

## 2. Materials and Methods

### 2.1. Experimental Materials

In this research, the raw materials of SA were obtained from Sichuan Neautus Traditional Chinese Medicine Co., Ltd. A herbal medicine roaster with online-type and noncontact temperature measurement system (ONTMS, Haishan Pharmaceutical Equipment Company Limited in Hangzhou) [25] was used for stir-baking SA samples. The ONTMS was connected to computer with software for temperature recording installed. The SA were put into roaster when the temperature increased to 220°C and then stir-baked to SBY, SBD, and SBC, respectively. SA samples were stir-baked repeatedly for three times to obtain three batches for each sample.

### 2.2. Color Measurement

#### 2.2.1. Computer Vision System

**Figure 1:**
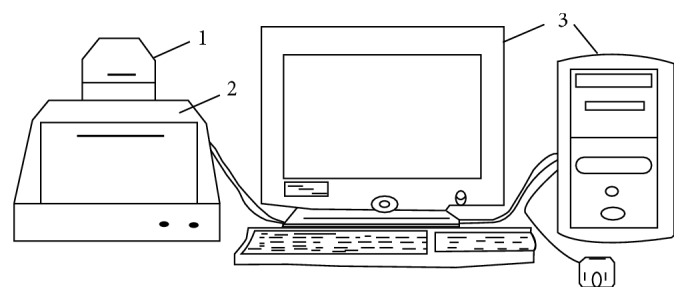
The CV system model used in this research. (1) A camera for capturing image, (2) a dark box with four fluorescent lights installed inside, and (3) a computer with imaging software installed.

Before the measuring, a computer vision system for image analysis was built (Figure 1) in order to standardize the procedure of capturing image. The image acquisition system used in this research consists of three components: RGB color camera (EOS 60D, Canon Inc., Japan) at resolution of 5184 × 3456 pixels (the camera parameters were as follows: shutter speed 1/80 s, manual operation mode, aperture Av F/10, ISO velocity 320, flash off, focal distance 60 mm, and lens: EF-S 60 mm f/2.8; a dark box with fluorescent lights was used; the dark box was applied to create an imaging chamber in order to avoid backscattering effects from other light sources; for the purpose of avoiding the undesirable reflections, internal stand walls were painted in gray matte color); four fluorescent lights (Philips Master TL-D 90 De Luxe 18 W/965) for illumination at a 45° angle to sample and with a color temperature of 6500 K; and a computer installed with software for image processing. The camera was calibrated by customizing the white balance using a white card (White Balance Card, 21.59 × 27.94 cm, X-rite) before capturing images.

During the measuring, the light was turned on for 30 minutes before capturing image to obtain stable light source. Each sample was placed manually on a white background at a distance of 40 cm to the camera. The image was captured using automatic remote shooting software in the computer linked with the camera. All the images acquired were stored in the computer and used for further analysis.

#### 2.2.2. Image Analysis

**Figure 2:**
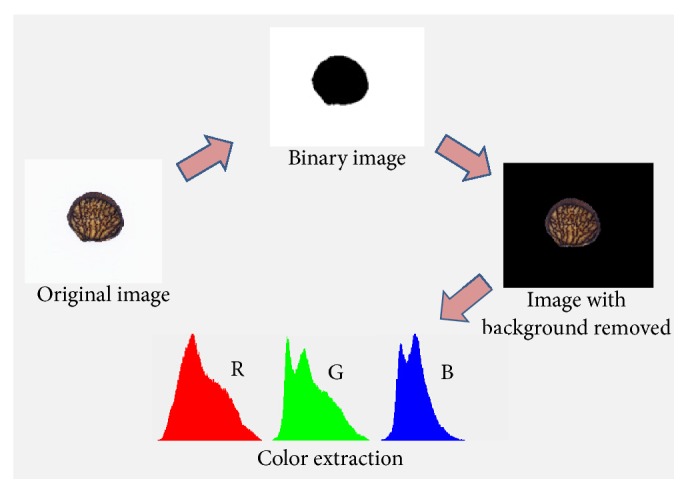
The procedure of image processing for SA images.

The image acquiring software used for color extraction was developed by our laboratory and introduced in detail in our previous work [26, 27]. The image process followed an appropriate procedure (Figure 2). Firstly, the background was removed from tested images. Then, objects were separated (segmentation was made), which means that the image was divided into parts corresponding to individual objects visible on the image (areas representing herbal medicine). Finally, the software had developed a function calculating the average value of RGB of all pixels, and then CIE *L* ^*∗*^ *a* ^*∗*^ *b* ^*∗*^ transformations have been implemented. 50 randomly chosen samples from each group were imaged, respectively, and the parameters of R, G, and B and *L* ^*∗*^, *a* ^*∗*^, and *b* ^*∗*^ were obtained for analysis. All the values of these parameters were transformed to the range of 0~255 before analyzing.

### 2.3. Electronic Nose

An E-nose system (FOX-4000, Alpha M.O.S., France) was used, which consists of a sampling apparatus, a detector unit containing an array of sensors, air generator equipment, HS-100 autosampler, and pattern recognition software (Alpha M.O.S., Version 2012.45) for data recording. The sensor array used was composed of 18 metal oxide semiconductors (MOS) chemical sensors, divided into chambers as three types: T (T30/1, T40/2, T40/1, TA/2, and T70/2), P (P10/1, P10/2, P40/1, PA/2, P30/1, P40/2, and P30/2), and LY (LY2/LG, LY2/G, LY2/AA, LY2/GH, LY2/gCTL, and LY2/gCT).

**Figure 3:**
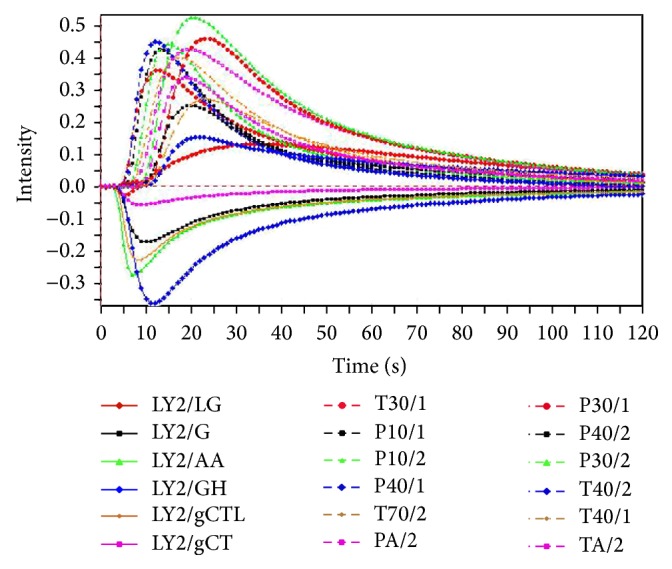
Typical sensor responses of E-nose during the measurement.

Samples were crushed and filtered through a 50-mesh sieve (inside diameter 355 *μ*m ± 13 *μ*m) and were accurately weighed 1.0 g and placed in 20 mL sealed headspace vials before being loaded into the autosampler tray. In the testing process, synthetic dry air was pumped into the sensor chambers at a constant rate of 150 mL/min* via* an air transformer connected to a syringe during the measurement process. Then, 1500 *μ*L of headspace air was automatically injected into E-nose by a syringe and flow-injected into the carrier gas flow. The injection rate was 1500 *μ*L/s, and incubation temperature was maintained at 50°C. The incubation time was set to 1080 s, and the time between injections was set to 600 s.  Figure 3 shows the typical sensor responses for the sample of RAW. Finally, the maximum response points that were automatically recorded for each of the 18 sensors were used as the output values. Each group of the samples has three batches, and each batch sample was measured three times; thus, each group obtained nine groups of data. Based on the method mentioned above, good repeatability was investigated and shown in Table 1.

### 2.4. Electronic Tongue

A commercial E-tongue (*α*Astree, Alpha M.O.S., France) introduced in previous reports was employed in this research, which consisted of 7 cross-selective potentiometric sensors designated as ZZ, AB, GA, BB, CA, DA, and JE, an Ag/AgCl reference electrode (Metrohm, Ltd.), a mechanical stirrer (Metrohm, Ltd.), a 16-position sample changer, and an interface electronic module for signal amplification and analog-to-digital conversion (Alpha M.O.S.) [17, 28]. The E-tongue was connected to a computer with the Astree II software (Alpha M.O.S., Version 2012.45) installed.

The functionality of the sensors was proven by a conditioning, calibration, and diagnosis procedure performed before every measurement. Within the conditioning and calibration phase, the sensors were rehydrated and the stability of the sensor response was tested using 0.01 mol/L hydrochloric acid. The diagnostic step measured by all the sensors could distinguish between 0.01 mol/L hydrochloric acid, 0.01 mol/L sodium-L-glutamate, and 0.01 mol/L sodium chloride solution.

**Figure 4:**
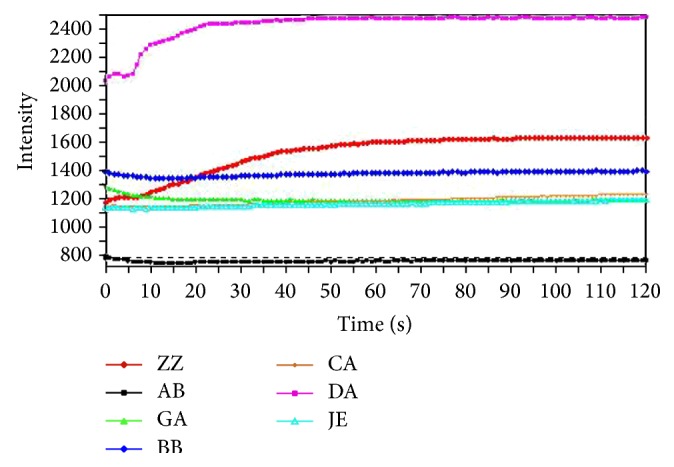
Typical sensor responses of E-tongue during the measurement.

Samples were crushed and filtered through a 50-mesh sieve (inside diameter 355 *μ*m ± 13 *μ*m) before being detected. 5.0 g of sample was accurately weighed and placed into a stoppered conical flask. Then, 100 mL of pure water was added and the extractives were refluxed for 1 hour and allowed to cool. After filtration, the filtrate was diluted to 250 mL. Then, 80 mL of liquid was placed in a beaker and loaded into the autosampler tray. All the samples were analyzed by E-tongue for 120 s.  Figure 4 shows the typical sensor responses for the sample of RAW, and the stable sensor responses between 100 s and 120 s were transformed to an average value, which was used as the output. The sensors were rinsed with deionized water after every analysis cycle. Using well-conditioned sensors, each sample was usually tested ten times by a rotation procedure (the first round of measurements of all samples was completed before the next round of measurements was started). For data processing, the last three rounds of measurement were used. Each group of the samples has three batches; thus finally each group obtained nine groups of data. Based on the method mentioned above, good repeatability was investigated and shown in Table 2.

### 2.5. Components Determination

#### 2.5.1. 5-Hydroxymethyl Furfural (5-HMF)

A modified method is a reference for the 5-HMF content determination in this research [29]. The HPLC analysis for content determination was performed in a Shimadzu LC-2010A system with a Phenomenex C18 column (250 mm × 4.6 mm, 5 *μ*m). The wavelength was set to 283 nm. Elution was performed at a flow rate of 1.0 mL·min^−1^, using the mobile phase consisting of a mixture of acetonitrile (8%) and water (92%). And the column temperature was maintained at 25°C.

#### 2.5.2. Arecoline (ARE)

ARE was determined according to the method recorded in Chinese Pharmacopoeia [30]. HPLC measurements were performed using a Shimadzu LC-2010A system with a Swell Chromstar SCX column (250 mm × 4.6 mm, 5 *μ*m), and the wavelength was set to 215 nm. The mobile phase consists of a mixture of acetonitrile (55%) and phosphoric acid solution (45%; the phosphate was diluted from 2 mL to 1000 mL and pH was adjusted to 3.8 using ammonia), the flow rate was set to 1.0 mL·min^−1^, and the column temperature was maintained at 25°C.

### 2.6. Statistical Processing

Principal component analysis (PCA) and discriminant factor analysis (DFA), as the two common multivariate analysis methods, have been widely used for classification [9, 31, 32]. In this research, PCA and DFA were performed using the Alpha M.O.S. statistical software, and a one-way analysis of variance (ANOVA) was conducted using SPSS 17.0.

## 3. Results

### 3.1. Color Analysis

Comparing the ability of color parameters to distinguish between different groups of SA, the ANOVA and Duncan multiple comparison method was employed and the mean values between any two of the four groups were compared. The significance level *p* for parameters R, G, *L* ^*∗*^, and *b* ^*∗*^ is *p* < 0.0001, *p* < 0.005 is for parameter B, and *p* < 0.05 is for parameter *a* ^*∗*^, representing the significant difference that appeared among the mean values of the color parameters.

**Figure 5:**
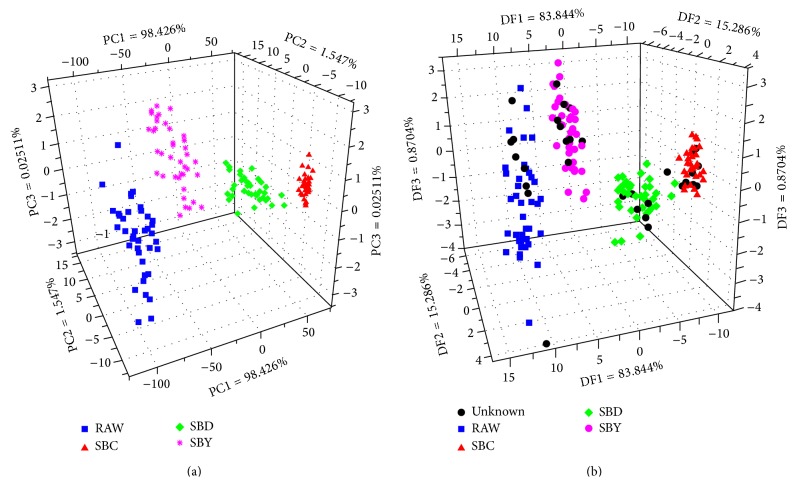
PCA and DFA scores plots for discriminating SA groups according to CV.

According to the color parameters that were acquired by CV, 6 parameters (R, G, B, *L* ^*∗*^, *a* ^*∗*^, and *b* ^*∗*^) were used as the inputs of PCA.  Figure 5(a) shows a three-dimensional scores plot of the first three principal components (PC1 = 98.426%; PC2 = 1.547%; PC3 = 0.025%). As can be seen, the samples representing four groups can be discriminated clearly. In addition, the DFA model (Figure 5(b)) was developed for recognition. In this model, 40 samples of each group were used as the calibrating group to train the model, and the remaining 10 unknown samples were injected into the model used as the testing group. As can be seen, the samples were divided into four groups, and after the unknown samples were projected into the model, good recognition was achieved, and all the unknown samples were correctly recognized.

According to the analysis of PCA and DFA, the information of the discreteness can be obtained from Figure 5. And combining with the standard deviation test, the discretization level of RAW > SBY > SBD > SBC was achieved (Table 3), representing the fact that the color of SA becomes more homogeneous after stir-baking.

### 3.2. Electronic Sensory Response of E-Nose

**Figure 6:**
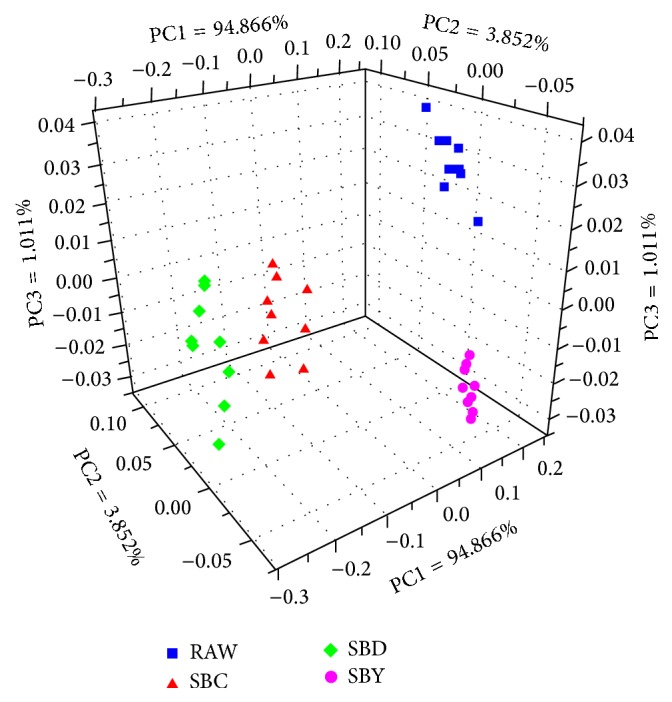
PCA scores plots for discriminating SA groups according to E-nose.

According to the electronic signals that were acquired by E-nose, sensor values (18 variables) were used as the inputs of PCA.  Figure 6 shows a three-dimensional scores plot of the first three principal components (PC1 = 94.866%; PC2 = 3.852%; PC3 = 1.011%). As shown in Figure 6, the samples were divided into four groups that represent RAW, SBY, SBD and SBC, respectively, and the samples perform clear discrimination among each group.

### 3.3. Electronic Sensory Response of E-Tongue

**Figure 7:**
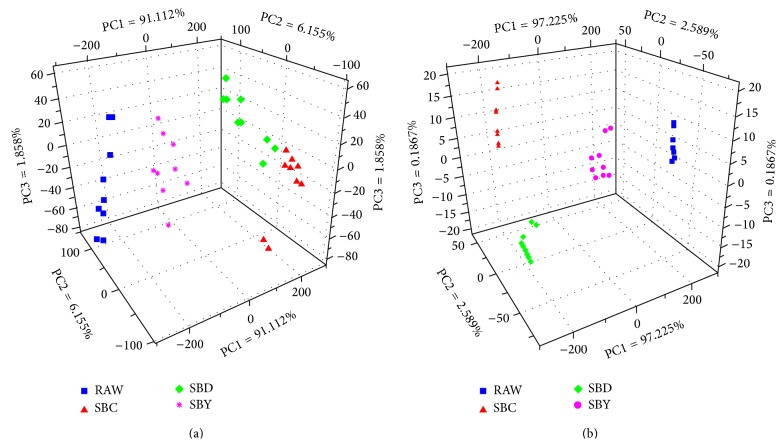
PCA scores plots for discriminating SA groups according to E-tongue.

According to the electronic signals that were acquired by E-tongue, sensor values (7 variables) were used as the inputs of PCA.  Figure 7(a) shows a three-dimensional scores plot of the first three principal components (PC1 = 91.112%; PC2 = 6.155%; PC3 = 1.858%). As shown in Figure 7(a), the samples were divided into four groups that represent RAW, SBY, SBD and SBC, respectively; the samples belonging to groups RAW and SBY can be discriminated vastly, but groups SBD and SBC cannot be separated from each other clearly. Additionally, all groups performed obvious dispersion.

In order to achieve better discrimination according to E-tongue, sensor selection was explored. To compare the discriminating ability of particular sensors, ANOVA was employed first [31]. The result shows that the significance level *p* for each sensor is <0.0001, representing the notion that the significant difference that appeared among the mean values of the SA groups was detected by any of the 7 sensors. But the discriminating ability of these sensors was obtained based on the test for homogeneity of variance; the corresponding *F* value of each sensor is DA (719.747) > ZZ (603.228) > CA (143.882) > JE (38.822) > AB (27.587) > BB (13.211) > GA (7.112). Thus, the first three sensors, DA, ZZ, and CA, were chosen as the new variables, with the best discriminating ability.  Figure 7(b) shows a three-dimensional scores plot of the first three principal components (PC1 = 97.225%; PC2 = 2.589%; PC3 = 0.1867%) of PCA based on these three variables. As can be seen, the samples belong to four groups which are discriminated obviously among each other.

### 3.4. Correlations between Instrumental Values and Components

#### 3.4.1. The Difference of 5-HMF and ARE in Semen Arecae Groups

**Figure 8:**
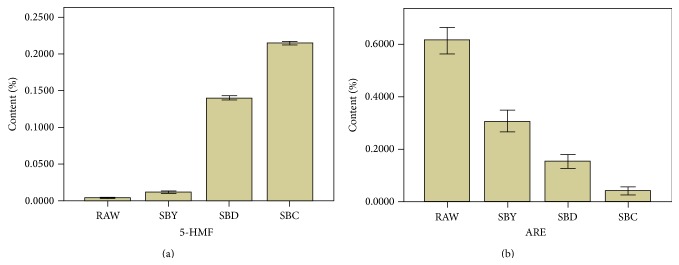
The content of 5-HMF and ARE in SA and its processed products (*n* = 3).

Using the contents of 5-HMF and ARE as variables, the ANOVA and Duncan multiple comparison method was applied to compare the mean values between any two of the four groups. The significance level *p* for each variable is *p* < 0.001, representing the notion that a significant difference appears among the mean values of the contents. The results show that, combining with the stir-baking degree deepening, the content of 5-HMF is increasing vastly (Figure 8(a)), but the content of ARE is decreasing significantly (Figure 8(b)). That indicated that 5-HMF and ARE can be the indicators of stir-baking degree.

#### 3.4.2. Correlations

Firstly, the original data obtained by CV, E-nose, and E-tongue were transformed to the mean values. Then, the factors representing integrated indexes of color parameters and electronic sensory values were extracted, and the correlations between these factors and components content were investigated. For CV, one factor (FAC1, 87.805%) was extracted; for E-nose, one factor (FAC1, 93.974%) was extracted; and two factors (FAC1, 69.809%; FAC2, 14.849%) were extracted for E-tongue.

Pearson's correlation test was used to analyze the correlation between components and extracted factors. According to the result (Table 4), the contents of 5-HMF and ARE are significantly correlated with color parameters and sensor responses of E-nose (*p* < 0.001). And also, these two components are significantly correlated with the FAC1 of E-tongue, but no correlations are performed with the FAC2. Because only 69.809% of the total cumulative variance can be explained by FAC1, further correlations between components and E-tongue sensors were explored. The result (Table 5) indicates that sensor responses of ZZ, AB, BB, CA, DA, and JE were closely related to 5-HMF and ARE, but there are no correlations between sensor GA and components.

## 4. Discussion

It is reported that Maillard Reaction universally exists in heating procedure of food and herb medicine, and it is the main course leading to the changes of color and flavor of the object [33–35]. Additionally, stir-baking of SA is a process combining with this reaction that has been proved by our original research [29]. So, in this research, the CV, E-nose, and E-tongue were applied to detect the changes of color and flavor in SA, to achieve the discrimination of SA and its processed products. To the best of our knowledge, this research is the first report regarding rapid and accurate discrimination of SA and its processed products by using CV, E-nose, and E-tongue.

As one of the most important indices, the color is often used for the evaluation of many herb medicines, which is usually evaluated by trained specialists. However, the evaluation of color is easily influenced by environment, illumination, subjective visual difference, and so forth. Thus, different people might give different evaluations for the same object. In contrast, CV is a rapid, nondestructive, nonexpansive, efficient, repeatable, precise, and consistent technique, and it can be used for color analysis of the sensorial attributes of herb medicine.

E-tongue and E-nose, which are composed of an array of cross-responsive sensors, are designed to detect an integral response of all the related chemicals in odor or liquid instead of a particular compound [20, 36]. They treat the mixtures as a single analyte and collect a combined sensors response. Thus, products with similar chemical substances in odor or liquid generally result in similar sensor response patterns (similar “fingerprints”), whereas products with different chemical substances show differences in their patterns (different “fingerprints”) [37]. Volatile compounds in odor and chemical substances in liquid are closely related to the chemical substances that exist in the materials. The particular sensors responses of E-nose and E-tongue can be obtained, because the components in SA and its processed products are different [38], as well as in odor and liquid. The difference in sensor response patterns is the key to discriminate SA groups in this research.

As a key intermediate of Maillard Reaction, 5-HMF has been evaluated as indicator of the severity of heat treatment or length of storage in several heating products [39–41]. Moreover, the main known ingredient of SA is arecoline, which is considered the effective constituent [42]. So these two components were chosen as the indicators for components analysis in our present research. A vast difference of these components' content among SA and its different processed products was achieved, and it indicated that 5-HMF and ARE can be the indicators of stir-baking degree of SA. In addition, the close correlations between instrumental characteristics (color, electronic sensory responses of E-nose and E-tongue) and the components were obtained. The close correlations represent the notion that the objective instrumental characteristics can reflect the quality of SA and its processed products.

In conclusion, intelligent sensory technologies including CV, E-nose, and E-tongue were applied to detect SA samples, and a novel method for the discrimination of SA and its processed products was developed coupled with chemometrics. The result indicates that, based on the objective instrumental values obtained by these three technologies, SA and its processed products were discriminated clearly coupled with chemometrics, such as PCA and DFA. Moreover, better discrimination based on E-tongue can be acquired after a sensor selection by ANOVA. Additionally, obvious changes of indicative components including 5-HMF and ARE were performed by the content determination, and the close correlations with color and sensory responses of E-nose and E-tongue were acquired. The close correlations represent the notion that the CV, E-nose, and E-tongue have a very good potential for quality evaluation of SA and its processed products. Therefore, the analytical method proposed based on CV, E-nose, and E-tongue is rapid, objective, and simple, and it can successfully discriminate the SA and its different processed products.

## Figures and Tables

**Table 1:** The repeatability based on the detective method of E-nose (*n* = 6).

Sensor	RSD (%)
LY2/LG	1.41
LY2/G	1.22
LY2/AA	1.01
LY2/GH	1.46
LY2/gCTL	1.14
LY2/gCT	1.14
T30/1	0.57
P10/1	0.38
P10/2	0.66
P40/1	0.62
T70/2	0.47
PA/2	0.75
P30/1	0.70
P40/2	0.97
P30/2	1.59
T40/2	0.70
T40/1	0.98
TA/2	0.73

**Table 2:** The repeatability based on the detective method of E-tongue (*n* = 6).

Sensor	ZZ	AB	GA	BB	CA	DA	JE

RSD (%)	0.51	0.29	1.39	1.65	0.46	0.16	0.72

**Table 3:** Standard deviation test result of the color parameters values.

Group	R	G	B	*L* ^*∗*^	*a* ^*∗*^	*b* ^*∗*^
RAW	5.55	5.77	5.88	5.73	0.85	1.31
SBY	6.93	6.64	6.03	7.10	0.77	1.54
SBD	5.90	3.78	2.48	4.90	0.80	1.75
SBC	2.16	2.02	2.65	2.55	0.21	0.39

**Table 4:** Pearson's correlations between components and extracted indexes.

	CV	E-nose	E-tongue	
	FAC1	FAC1	FAC1	FAC2
5-HMF				
Coefficients	−0.964	0.965	−0.906	−0.09
*p*	<0.001	<0.001	<0.001	0.78
ARE				
Coefficients	0.969	−0.903	0.952	0.007
*p*	<0.001	<0.001	<0.001	0.984

**Table 5:** Pearson's correlations between components and E-tongue sensors.

	ZZ	AB	GA	BB	CA	DA	JE
5-HMF							
Coefficients	−0.946	−0.681	<0.01	0.603	0.775	−0.972	0.885
*p*	<0.001	0.015	0.999	0.038	0.003	<0.001	<0.001
ARE							
Coefficients	0.898	0.622	−0.107	−0.712	−0.975	0.923	−0.976
*p*	<0.001	0.031	0.74	0.009	<0.001	<0.001	<0.001

## Abbreviations

SA: Semen Arecae
RAW: Raw material
SBY: Stir-baking to yellow
SBD: Stir-baking to dark brown
SBC: Stir-baking to carbon dark
CV: Computer vision
E-nose: Electronic nose
E-tongue: Electronic tongue
5-HMF: 5-Hydroxymethyl furfural
ARE: Arecoline
PCA: Principal component analysis
DFA: Discriminant factor analysis.
